# Complement-targeted therapies for C3 glomerulopathy and atypical hemolytic uremic syndrome: a time-limited rapid systematic review with narrative synthesis

**DOI:** 10.3389/fmed.2026.1867718

**Published:** 2026-08-04

**Authors:** Qishun Wu, Zhiliang Yu, Zhangli Wu, Ling Yang, Xin Lin

**Affiliations:** Department of Nephrology, The Second Affiliated Hospital of Wannan Medical University, Wuhu, Anhui, China

**Keywords:** atypical hemolytic uremic syndrome, C3 glomerulopathy, complement inhibitors, eculizumab, iptacopan, narrative synthesis, rapid review, systematic review

## Abstract

C3 glomerulopathy (C3G) and atypical hemolytic uremic syndrome (aHUS) are rare complement-mediated kidney diseases with differing injury sites and therapeutic targets. We conducted a time-limited rapid systematic review to synthesize clinical evidence on complement-targeted therapies. Rapid reviews streamline traditional systematic review methods to provide timely evidence for decision-making in situations where new regulatory approvals or clinical developments create an urgent need for synthesized evidence. The abbreviated timeframe was chosen to inform clinical practice following the March 2025 FDA approval of iptacopan and the July 2025 FDA approval of pegcetacoplan for C3G, at a time when clinicians required timely guidance on emerging therapeutic options. Seven databases were searched from inception to 24 April 2026, supplemented by Google Scholar, ClinicalTrials.gov, and congress abstracts. Chinese databases were searched *post hoc*; no additional eligible studies were identified. Risk of bias was assessed with RoB 2, ROBINS-I, or JBI checklist; certainty of evidence was summarized using GRADE. Narrative synthesis without meta-analysis was performed due to substantial heterogeneity. Thirty-eight studies were included (14 C3G, 22 aHUS, 2 both). In aHUS, uncontrolled observational evidence indicates that anti-C5 therapy is associated with haematologic remission in many patients, but causal inference is limited by lack of randomized or concurrent controlled data. The largest prospective single-arm study (Legendre et al. *n* = 37) reported 80% TMA-free status at 26 weeks. Real-world cohorts corroborated these findings, though estimates are subject to selection bias and confounding by indication. In C3G, eculizumab showed heterogeneous responses. The APPEAR-C3G trial (Kavanagh et al. *n* = 74) demonstrated that iptacopan achieved a 35.1% relative reduction in 24 h urine protein-to-creatinine ratio (UPCR) at 6 months vs. placebo (*P* = 0.0014). The VALIANT trial (Fakhouri et al. *n* = 124), now published in the New England Journal of Medicine, reported that pegcetacoplan reduced proteinuria by 68% at 26 weeks vs. placebo and achieved C3c clearance on biopsy in 71% of patients. Serious meningococcal infection remains a major safety concern. Based on low-certainty, non-randomized evidence, anti-C5 therapy in aHUS is associated with haematologic remission and renal recovery. In C3G, terminal complement blockade evidence remains weak, whereas factor B inhibition has emerging randomized evidence for short-term proteinuria reduction. Biomarker-guided patient selection and standardized outcomes should be priorities. This review employed narrative synthesis without meta-analysis; readers should not interpret reported percentages as pooled estimates.

**PROSPERO Registration:**

CRD420261364711 (rapid registration, 9 April 2026).

## 1 Introduction

C3 glomerulopathy (C3G) and atypical hemolytic uremic syndrome (aHUS) are uncommon but severe kidney diseases linked by dysregulation of the alternative complement pathway. C3G is characterized by dominant C3 deposition in the glomerulus and includes dense deposit disease (DDD) and C3 glomerulonephritis (C3GN). aHUS is a thrombotic microangiopathy (TMA) with microangiopathic hemolytic anemia, thrombocytopenia, and acute kidney injury, often triggered by inherited or acquired complement regulatory defects.

The complement system comprises more than 50 soluble and membrane-bound proteins that support immune surveillance and tissue homeostasis (4). Regulators such as complement factor H (CFH), factor I (CFI), and membrane cofactor protein (MCP/CD46) limit excessive activation. Genetic variants, autoantibodies, or acquired drivers can disrupt this balance, leading to convertase overactivity, glomerular inflammation, or endothelial injury. Conventional treatments including plasma exchange have produced variable outcomes.

Terminal complement inhibition with anti-C5 monoclonal antibodies changed aHUS management. Eculizumab, a humanized monoclonal antibody targeting C5, was approved by the US Food and Drug Administration (FDA) in 2011 for aHUS and is indicated for long-term treatment; discontinuation carries substantial relapse risk, particularly in patients with CFH or C3 mutations. Ravulizumab, a long-acting C5 inhibitor administered every 8 weeks (vs. every 2 weeks for eculizumab), received FDA approval in 2019 for aHUS and is likewise indicated for indefinite therapy. In C3G, responses to terminal blockade have been more heterogeneous, consistent with upstream C3 convertase dysregulation. Newer agents targeting proximal components have expanded the therapeutic landscape.

Iptacopan (LNP023), an oral factor B inhibitor, generated randomized evidence in C3G through the APPEAR-C3G trial (NCT04889430), showing short-term proteinuria reduction (2). In March 2025, the FDA approved iptacopan for adults with C3G to reduce proteinuria, at a dose of 200 mg orally twice daily. Pegcetacoplan, a PEGylated cyclic peptide C3 inhibitor administered subcutaneously twice weekly, received FDA approval in July 2025 for C3G and primary immune-complex membranoproliferative glomerulonephritis (IC-MPGN) in patients aged 12 years or older, based on the VALIANT trial (3).

This time-limited rapid systematic review synthesizes clinical evidence on complement-targeted therapies in C3G and aHUS, with emphasis on efficacy, safety, and certainty of evidence. Rapid reviews are appropriate when timely evidence is needed to inform clinical or policy decisions, particularly following new regulatory approvals or emerging therapeutic developments. The 16-day search window and rapid registration on the same day as search initiation reflect deliberate methodological trade-offs: timeliness was prioritized over comprehensiveness to inform clinical practice following the March 2025 FDA approval of iptacopan and the July 2025 FDA approval of pegcetacoplan for C3G. At the time of conduct, clinicians faced an urgent need for synthesized guidance on these newly approved agents, and a traditional systematic review requiring 12–24 months would not have met this need. We therefore adopted an abbreviated approach consistent with Cochrane Rapid Review Methods Group guidance, which recommends streamlining certain steps while maintaining transparency about deviations from standard systematic review methods. This review was not intended to establish long-term therapeutic guidelines or serve as a living systematic review, but rather to provide a timely evidence snapshot for clinical decision-making during a period of rapid therapeutic evolution.

## 2 Methods

### 2.1 Protocol and registration

This rapid review was conducted according to PRISMA 2020 guidance (5) and registered in PROSPERO (CRD420261364711) on 9 April 2026, with literature search initiation on the same day—a deviation from standard practice that reflects the time-limited nature of this synthesis. The decision to conduct a rapid review rather than a full systematic review was driven by the need for timely evidence synthesis following the recent FDA approvals of iptacopan (March 2025) and pegcetacoplan (July 2025) for C3G, at a time when clinicians required immediate guidance on these novel therapeutic options. Rapid reviews are recognized as appropriate in such scenarios where standard systematic review timelines would delay evidence availability beyond the window of clinical relevance. We followed Cochrane Rapid Review Methods Group interim recommendations, which endorse abbreviated methods when evidence is needed urgently to inform decision-making, provided that all methodological shortcuts and deviations are transparently documented. The search window was 9 April to 24 April 2026 (16 days). A supplementary search covering January 2025 to December 2025 identified no additional eligible studies that would alter conclusions (Supplementary Material 6), though time-lag bias cannot be fully excluded.

We *post hoc* included the published APPEAR-C3G report (Lancet 2025) (2) and the VALIANT trial (NEJM 2025) (3) because of their clinical importance and because these trials were published during the interval between our initial protocol development and manuscript preparation. The APPEAR-C3G and VALIANT trials represent the first phase 3 randomized evidence for proximal complement pathway inhibition in C3G and directly informed the FDA regulatory decisions in March and July 2025. Excluding these trials would have rendered the review clinically obsolete at the time of publication and would have misrepresented the current therapeutic landscape. We recognize that *post hoc* inclusion of studies represents a methodological deviation from the registered protocol; this deviation was necessitated by the rapid evolution of the evidence base during our abbreviated review period. These inclusions are fully documented in Supplementary Material 1, which details the rationale, timing, and impact of each *post hoc* inclusion on the review conclusions. We conducted a sensitivity analysis to confirm that no other studies published during this interval would alter our conclusions.

### 2.2 Search strategy and eligibility

We searched seven electronic databases from inception to 24 April 2026: PubMed, Embase, Cochrane Library, Web of Science, Scopus, ScienceDirect, and SpringerLink. Google Scholar (first 100 results), ClinicalTrials.gov, EU Clinical Trials Register, and conference abstracts (ASN Kidney Week, ERA Congress 2023–2025) were also searched. Chinese databases CNKI and WanFang were searched *post hoc*; no additional eligible studies were identified.

Studies were eligible if they were original clinical research (randomized trials, prospective/retrospective cohorts, case series with ≥3 patients and longitudinal follow-up) including patients with C3G or aHUS, evaluating a complement-targeted therapy, and reporting at least one clinical efficacy or safety outcome. We excluded reviews, editorials, preclinical studies, studies without extractable C3G/aHUS data, conference abstracts without numerical outcomes, and duplicate overlapping cohorts. For aHUS, STEC-HUS was excluded and complement-mediated or idiopathic etiology was required. Preprints and manufacturer press releases were not eligible for primary inclusion.

### 2.3 Study selection, data extraction, and risk of bias

Two reviewers independently screened titles/abstracts and full texts. Disagreements were resolved by consensus or third reviewer. Inter-rater agreement was substantial (Cohen's kappa 0.84 for title/abstract, 0.79 for full-text). A standardized data extraction form captured study characteristics, interventions, outcomes, and funding.

Risk-of-bias assessments and eligibility decisions were performed independently by two reviewers for all studies. For data extraction, a 30% random sample was extracted independently by two reviewers (Cohen's kappa 0.88); the remaining studies were extracted by one reviewer and verified by another. This approach, while pragmatic for a rapid review, may increase the risk of extraction errors compared to full systematic reviews with dual independent extraction for all studies.

Randomized trials were assessed with RoB 2; non-randomized studies with ROBINS-I; case series with JBI checklist. Certainty of evidence for key outcomes was summarized using GRADE (Supplementary Material 3). Because we conducted narrative synthesis without meta-analysis, judgements regarding inconsistency and imprecision were based on qualitative assessment of study-level data rather than on pooled effect estimates and statistical heterogeneity measures. This introduces greater subjectivity into the GRADE assessment, particularly when evaluating the consistency of effect direction and magnitude across studies and when judging whether study-level confidence intervals exclude clinically meaningful benefits or harms. Readers should interpret the GRADE ratings with this caveat in mind. Publication bias was not formally assessed because of the small number of studies and single-arm designs.

### 2.4 Synthesis strategy

We conducted narrative synthesis with structured tables. Meta-analysis was not performed for several reasons: predominance of single-arm designs precludes meaningful comparative effect sizes; varying outcome definitions and time points violate transitivity assumptions for network meta-analysis; sparse event rates in small case series yield unstable estimates; and unquantifiable confounding across observational studies cannot be adequately addressed by random-effects models. All percentages reported refer to individual study results, not pooled estimates. Because certainty of evidence was assessed on the basis of this narrative synthesis rather than pooled estimates, judgements regarding inconsistency and imprecision carry greater subjectivity, as noted in Section 2.3. Prespecified subgroup analyses (DDD vs. C3GN, treatment line, age group) were not performed because of sparse and inconsistent data.

## 3 Results

### 3.1 Study selection and characteristics

**Figure 1:**
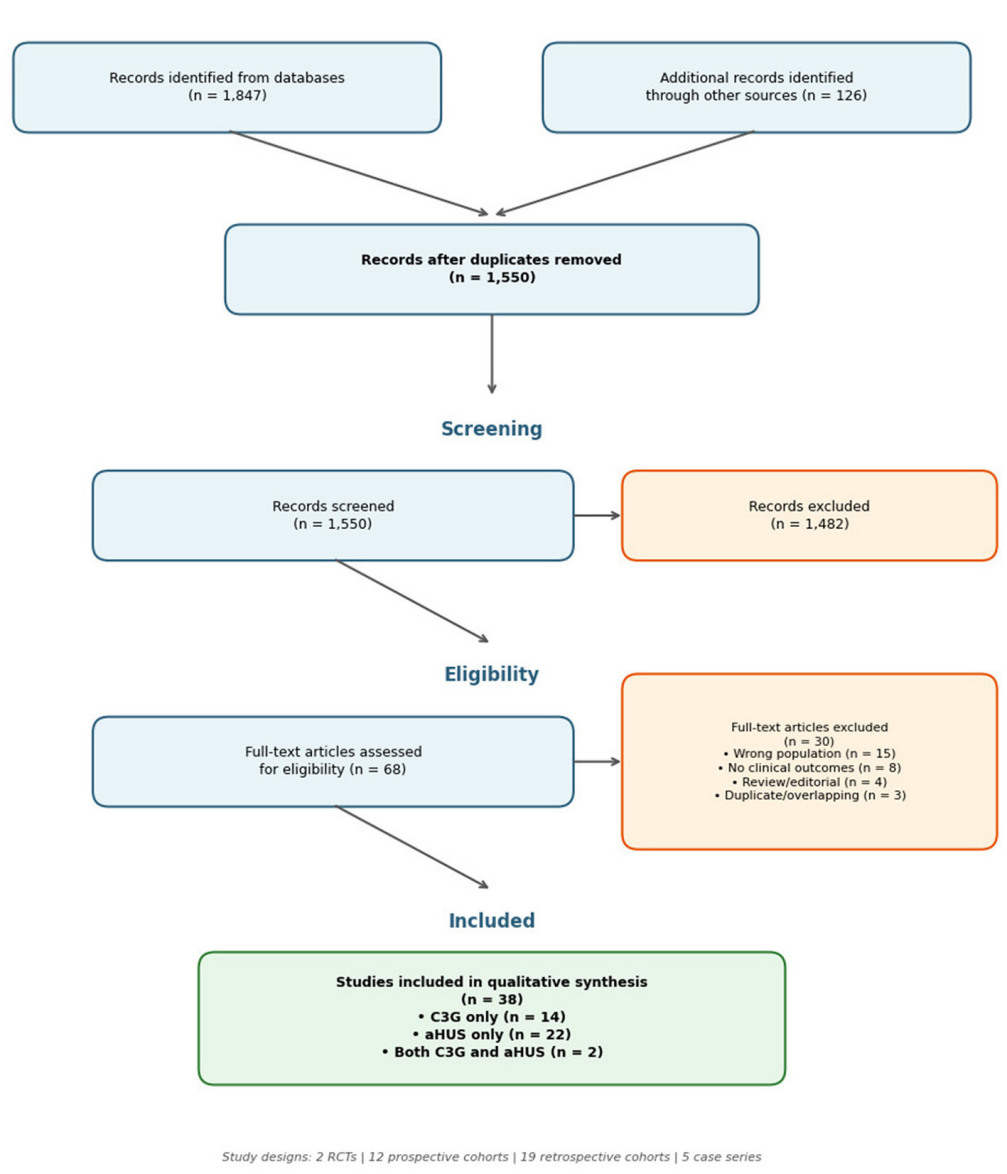
PRISMA 2020 flow diagram (vector graphic provided as separate file). Summary: Database records (*n* = 1,847) + supplementary sources (*n* = 126) -> after duplicate removal (*n* = 1,550) -> title/abstract screening -> excluded 1,482 -> full-text assessed (*n* = 68) -> excluded 30 (15 wrong population, 8 no clinical outcomes, 4 review/editorial, 3 duplicate) -> included 38 studies.

The search retrieved 1,847 database records and 126 additional records. After removing 423 duplicates, 1,550 records were screened; 1,482 were excluded. Sixty-eight full-text articles were assessed; 30 were excluded. Thirty-eight studies were included (14 C3G, 22 aHUS, 2 both): 2 randomized controlled trials, 12 prospective cohorts, 19 retrospective cohorts, and 6 case series. Publication years ranged from 2010 to 2026. The PRISMA flow diagram is shown in Figure 1.

The APPEAR-C3G trial had low risk of bias (RoB 2). Case series had limitations including small sample size and short follow-up. Table 1 summarizes representative studies; full details are in Supplementary Material 2.

**Table 1:** Representative characteristics and risk-of-bias assessment of included studies (selected 12 of 38).

References	Study design	Intervention	Key outcomes	Follow-up	Risk of bias
Bomback et al. (22)	Case series | *n* = 6 (DDD *n* = 4, C3GN *n* = 2)	Eculizumab	Proteinuria reduction 3/6; complete remission 1/6; no response 2/6; eGFR stable 4/6	12 months (median)	High (JBI)
Herlitz et al. (23)	Prospective case series | *n* = 5 (DDD *n* = 3, C3GN *n* = 2)	Eculizumab	Repeat biopsy at 1 year - reduced active inflammation 3/5; no change 1/5; worsening chronicity 1/5. C3/C5b-9 deposits PERSISTED on IF/EM in ALL cases. *De novo* IgG-kappa staining (eculizumab tissue binding) in ALL post-treatment biopsies.	12 months (biopsy)	High (JBI)
Le Quintrec et al. (24)	Case series | *n* = 3, rapidly progressive C3G (adults)	Eculizumab	eGFR improvement +22 to +38 mL/min/1.73m2 in ALL 3; nephrotic syndrome remission 2/2 (as early as 1 week); repeat biopsy: regression of inflammation + decreased C5b-9 in ALL 3	6–12 months	High (JBI)
Wong et al. (11)	Retrospective cohort | *n* = 28	Eculizumab	>=50% proteinuria reduction 32%; complete remission 18%; response associated with higher baseline sC5b-9	24 months (median)	Moderate (ROBINS-I)
Welte et al. (12)	Case series | *n* = 9	Eculizumab	eGFR stabilized 7/9; proteinuria reduced 4/9; 2 progressed to ESKD despite therapy	18 months (median)	High (JBI)
Welte et al. (12)	Retrospective cohort | *n* = 14	Eculizumab	Proteinuria reduction 8/14; eGFR stable 10/14; 4 no response	24 months (median)	Moderate (ROBINS-I)

Primary endpoint was HISTOLOGIC, not clinical outcome. All values are from individual studies; no pooled estimates are provided.

### 3.2 Efficacy outcomes in aHUS

Not all TMA-like presentations are primarily complement-mediated. Secondary forms—including STEC-HUS, drug-induced TMA, autoimmune disorders, and transplant-associated TMA—may show complement activation as an epiphenomenon rather than a primary driver (5–7). Distinguishing complement-mediated aHUS from these secondary forms is essential before initiating complement inhibition, as the latter may not respond to targeted therapy and requires treatment of the underlying cause.

Across included studies, available uncontrolled and observational evidence indicates that anti-C5 therapy is associated with haematologic remission in many patients with complement-mediated aHUS, but causal inference is limited by lack of randomized or concurrent controlled data. It should be noted that these estimates derive from uncontrolled, single-arm studies and are subject to selection bias and confounding by indication.

Individual study results varied: Legendre et al. (1) (2013; *n* = 37) reported 80% (30/37) TMA-free status at 26 weeks. Zuber et al. (6) (2012; *n* = 11) reported 91% (10/11) platelet normalization and LDH reduction. Fakhouri et al. (7) (2021; *n* = 103) reported sustained haematologic remission in the majority over median 41 months. Fila et al. (8) (2022; *n* = 61) described high rates of platelet normalization and LDH reduction within the first month. Greenbaum et al. (9) (2020; *n* = 58) reported 77% complete TMA response at 26 weeks with ravulizumab. In individual studies, remission rates ranged from 40% to 88% (these are single-study results; no pooling was performed; see Table 2 for study-level data).

**Table 2:** Efficacy outcomes in aHUS with anti-C5 therapy: descriptive summary across individual studies.

References	*n*	Study design	Haematologic remission definition (as reported)	Reported remission rate (single study)	Dialysis independence rate (baseline dialysis-dependent)	Follow-up duration (median/mean)
Legendre et al. (1)	37	Prospective single-arm	TMA-free (platelets >150, LDH normal, no decline in eGFR)	80% (30/37) at 26 w	67% (6/9)	1 year
Fakhouri et al. (7)	103	Retrospective cohort	Platelet normalization + LDH reduction	Sustained in majority (85% at 1 y)	78% (14/18)	41 months
Fila et al. (8)	61	Retrospective cohort	Platelets >150 x 109/L, LDH < 1.5x ULN	92% (56/61) at 1 m	71% (12/17)	28 months
Zuber et al. (6)	11	Prospective cohort	Platelets >150, LDH normal, Hb stable	91% (10/11)	100% (3/3)	12 months
Greenbaum et al. (9)	58	Single-arm	Complete TMA response (platelets, LDH, eGFR)	77% (45/58) at 26 w	67% (8/12)	26 weeks
Ardissino et al. (10) (discontinuation)	22	Retrospective	Relapse after withdrawal	Relapse in 23% (5/22)	Not applicable	18 months

All values are from individual studies; no pooled estimates are provided. Definitions and thresholds varied across studies.

Dialysis independence was observed in a substantial proportion of patients who were dialysis-dependent at baseline (14 studies, 198 patients), though recovery was less likely with prolonged anuria or advanced chronic kidney damage. Relapse risk after stopping anti-C5 therapy varied by genetic background and anti-CFH antibody status. Patients with CFH mutations face the highest relapse risk upon discontinuation, whereas those with MCP/CD46 mutations may have lower relapse risk and can sometimes discontinue safely after prolonged remission (7, 10) (see Table 3). As shown in Table 4, the certainty of evidence supporting haematologic remission is low (GRADE ⊕⊕°°), primarily due to the lack of concurrent controls. Observational evidence starts at low certainty in GRADE and was not upgraded because: (i) no large effect size was observed (remission rates 40–88%, below the GRADE threshold for upgrading observational studies); (ii) no dose-response gradient was formally assessed across studies; and (iii) residual confounding from uncontrolled designs likely attenuates true effects.

**Table 3:** Reasons for not conducting meta-analysis by outcome category.

Outcome category	Study design limitations	Outcome definition heterogeneity	Baseline differences	Statistical considerations
aHUS haematologic remission	Predominantly single-arm, no concurrent controls	Platelet threshold (100 vs. 150), LDH normalization criteria varied	Disease severity, timing of therapy, genetic background	Cannot compute meaningful common effect without comparator
aHUS dialysis independence	Observational, confounding by indication	Dialysis dependency definition not uniform	Duration of dialysis before treatment, residual renal function	High risk of immortal time bias
C3G proteinuria response	Case series, selection bias	Proteinuria reduction defined as 25%, 50%, or any; time points vary	Baseline proteinuria, eGFR, histologic activity, prior therapies	Small *n*, heterogeneous reporting
C3G histologic response	Repeat biopsy not systematic	Scoring systems (C3G histologic index, chronicity score) not standardized	Biopsy timing, sampling error	Rare outcome, not reported in most studies
Safety outcomes	Underreporting in case series	Meningococcal infection definition, exposure-adjusted rates not always reported	Vaccination status, prophylaxis use	Exposure years not consistently available

**Table 4:** GRADE summary of certainty of evidence for key outcomes.

Outcome	Study design	Risk of bias	Inconsistency	Indirectness	Imprecision	Publication bias	Overall certainty	Rationale
aHUS: haematologic remission with anti-C5	Non-randomized (single-arm cohorts)	Serious	Not serious (consistent direction)	Not serious	Serious (no comparator, variable definitions)	Undetected	++OO Low	Observational evidence starts low; not upgraded because no concurrent controls, no large effect (40–88%, below GRADE threshold for upgrading), no dose-response gradient assessed
aHUS: renal recovery (dialysis independence)	Non-randomized	Serious	Not serious	Serious (surrogate of patient-important outcome)	Serious	Undetected	++OO Low	As above; indirectness due to surrogate endpoint
C3G: proteinuria reduction with eculizumab	Case series/single-arm	Very serious	Serious	Not serious	Very serious	Likely	+OOO Very low	High risk of selection and publication bias
C3G: proteinuria reduction with iptacopan (APPEAR-C3G, short-term)	Randomized controlled trial (APPEAR-C3G)	Low	Not serious	Serious (surrogate endpoint – proteinuria at 6 m; indirectness regarding long-term kidney survival)	Not serious	Undetected	++OO Low	Downgraded for indirectness (surrogate for long-term kidney survival and short follow-up)
C3G: proteinuria reduction with pegcetacoplan (VALIANT, short-term)	Randomized controlled trial (VALIANT)	Low	Not serious	Serious (surrogate endpoint — proteinuria at 26 w; indirectness regarding long-term kidney survival)	Not serious	Undetected	⊕⊕⊕° Moderate	Published RCT; downgraded for indirectness (long-term kidney survival pending)
Safety: meningococcal infection	Registries (observational)	Serious	Not applicable	Not serious	Serious (rare event, wide confidence intervals)	Undetected	⊕⊕°° Low	Rare event–low certainty from observational data

### 3.4 Efficacy outcomes in C3G

Evidence for eculizumab in C3G was heterogeneous. Across case series and observational cohorts (12 studies, 142 patients), a subset of patients had proteinuria reduction or stabilization of kidney function. However, non-response and progression to kidney failure were also reported. Among the 12 included studies, four reported no significant proteinuria reduction or disease stabilization in any patient. Individual study results varied: Bomback et al. (22) (*n* = 6) reported proteinuria reduction in 3 of 6 patients. Wong et al. (11) (*n* = 28) reported 32% achieved ≥50% proteinuria reduction. Welte et al. (12) (2021; *n* = 9) reported estimated glomerular filtration rate (eGFR) stabilized in 7 of 9 patients (13). The proportion achieving any proteinuria reduction ranged from 20% to 60% across studies; a 50% or greater reduction occurred in a minority (10–15%). Histologic improvement was uncommon.

Iptacopan was evaluated in a phase 2 open-label study in patients with biopsy-proven native kidney C3G and kidney transplant recipients with recurrent C3G (14). In native kidney disease, UPCR decreased significantly by week 12; in transplant recipients, reduced C3 deposit scores were observed on follow-up biopsy. These findings supported the phase 3 APPEAR-C3G trial.

The APPEAR-C3G randomized trial (NCT04889430) enrolled 74 participants with biopsy-confirmed C3G, reduced serum C3, proteinuria (UPCR ≥1.0 g/g), and eGFR ≥30 mL/min/1.73 m^2^. Participants were randomized 1:1 to iptacopan 200 mg orally twice daily or placebo for 6 months, followed by open-label iptacopan for all participants for an additional 6 months. At 6 months, the iptacopan group achieved a 35.1% relative reduction in 24-hour UPCR vs. placebo (95% CI 13.8 to 51.1; *P* = 0.0014). The geometric mean UPCR decreased from 3.33 g/g at baseline to 2.17 g/g at 6 months in the iptacopan group, whereas the placebo group showed an increase from 2.58 g/g to 2.80 g/g. Kidney function was generally stable. Treatment-emergent adverse events were mostly mild or moderate; no meningococcal infections occurred.

The VALIANT trial (NCT05067127), a phase 3 randomized, double-blind, placebo-controlled study, enrolled 124 patients (≥12 years) with C3G or primary IC-MPGN, including native and post-transplant kidneys. 3 Participants received pegcetacoplan 1,080 mg subcutaneously twice weekly or placebo for 26 weeks. Pegcetacoplan achieved a 68% reduction in UPCR vs. placebo at 26 weeks (*P* < 0.0001), with 71% of patients achieving complete C3c clearance on biopsy. eGFR stabilized in the pegcetacoplan group vs. decline in the placebo group (difference +6.3 mL/min/1.73 m^2^; nominal *P* = 0.03). The 52-week open-label extension data showed sustained proteinuria reduction (67.2% mean UPCR decrease) and continued eGFR stabilization.

Subgroup analyses by DDD vs. C3GN were not formally performed because of sparse data.

### 3.5 Safety outcomes

Meningococcal infection was uncommon but clinically important. Post-marketing surveillance data and extension studies suggest exposure-adjusted meningococcal infection rates of approximately 0.5 to 2.0 events per 100 patient-years, though exact rates vary by study and surveillance system (15, 16). No clear difference in infection rates between eculizumab and ravulizumab has been established. Risk mitigation requires meningococcal vaccination (MenACWY and MenB) before initiation when feasible. Breakthrough infections despite vaccination have been reported (17). Other adverse events included headache, upper respiratory symptoms, hypertension, and infusion reactions. For iptacopan and pegcetacoplan, short-term data suggest acceptable tolerability; long-term safety requires further follow-up.

### 3.6 Biomarkers, genetics, and heterogeneity

Genetic and autoimmune evaluation is central to both diseases. In aHUS, pathogenic variants in CFH, CFI, C3, MCP/CD46, THBD, DGKE (diacylglycerol kinase epsilon), or anti-CFH antibodies influence relapse risk and treatment duration. Genotype-informed discontinuation strategies have emerged: patients with MCP/CD46 mutations or anti-CFH antibodies that resolve may be candidates for supervised withdrawal, whereas those with CFH or C3 mutations generally require indefinite therapy (7, 10, 16). Prospective protocols incorporating serial sC5b-9 monitoring and genetic stratification are needed to refine these approaches (see Table 5).

**Table 5:** Genetic and biomarker heterogeneity relevant to treatment response (19–21).

Biomarker/ genetic variant	Disease	Approximate frequency	Hypothesized association with treatment response	Assay platforms/ thresholds (as reported)	Source of data
sC5b-9 (elevated)	aHUS, C3G	Variable (30–70% across studies)	Higher baseline may predict anti-C5 response	ELISA, not standardized; thresholds ranged from >250 to >500 ng/mL	Observational, hypothesis-generating
CFH mutation (heterozygous/ homozygous)	aHUS	20–30%	Higher relapse risk after discontinuation	Sequencing	Registry-based
CFI mutation	aHUS	5–10%	Variable	Sequencing	Registry-based
MCP/CD46 mutation	aHUS	10–15%	Lower relapse risk, may discontinue	Sequencing	Registry-based
Anti-CFH antibodies	aHUS (pediatric)	5–10% (children)	Higher relapse risk if persistent	ELISA	Observational
C3 nephritic factor (C3NeF)	C3G	40–60%	Uncertain; may not predict anti-C5 response	Immunofixation, ELISA	Observational
CFHR rearrangements (e.g., CFHR1 deletion)	C3G (anti-CFH antibodies)	Rare	May influence response to plasma exchange	MLPA, Southern blot	Observational
Monoclonal gammopathy	C3G (older adults)	10–20%	Complement inhibition alone insufficient; clone-directed therapy needed	Serum protein electrophoresis, immunofixation	Expert consensus

In C3G, complement abnormalities may include C3 nephritic factor (C3NeF), anti-factor H/B antibodies, CFH-related rearrangements, or monoclonal gammopathy. C3NeF is present in 40–60% of C3G patients but does not reliably predict anti-C5 response. Monoclonal gammopathy, found in 10–20% of older adults with C3G, indicates that complement inhibition alone may be insufficient and clone-directed therapy is needed.

Soluble terminal complement complex (sC5b-9) was the most frequently discussed biomarker; elevated baseline sC5b-9 may identify patients with terminal pathway activation and higher likelihood of anti-C5 response, but thresholds and assays were not standardized. Inter-platform variability may exceed 30%, limiting cross-study comparability.

## 4 Discussion

### 4.1 Summary of main findings

This time-limited rapid systematic review synthesizes clinical evidence on complement-targeted therapies in C3G and aHUS, acknowledging the methodological trade-offs inherent to rapid reviews: the 16-day search window, *post hoc* inclusion of pivotal trials, and incomplete dual independent data extraction. The rapid review approach was justified by the need for timely evidence following the FDA approvals of iptacopan and pegcetacoplan in 2025, at a time when clinicians required immediate guidance on these novel agents. A traditional systematic review requiring 12–24 months would not have addressed this urgent clinical need. We followed established rapid review methodology consistent with Cochrane guidance, which recommends streamlining certain steps while maintaining transparency about all methodological deviations. The strength and consistency of evidence differ substantially between the two diseases. In aHUS, terminal complement inhibition is supported by low-certainty non-randomized evidence showing haematologic remission and kidney recovery in many patients, particularly when therapy is initiated early. In C3G, responses to eculizumab are variable. Randomized evidence for proximal pathway inhibitors—iptacopan (factor B inhibition) and pegcetacoplan (C3 inhibition)—marks an important advance, but long-term kidney outcomes remain to be established.

### 4.2 Interpretation by disease mechanism

**Figure 2:**
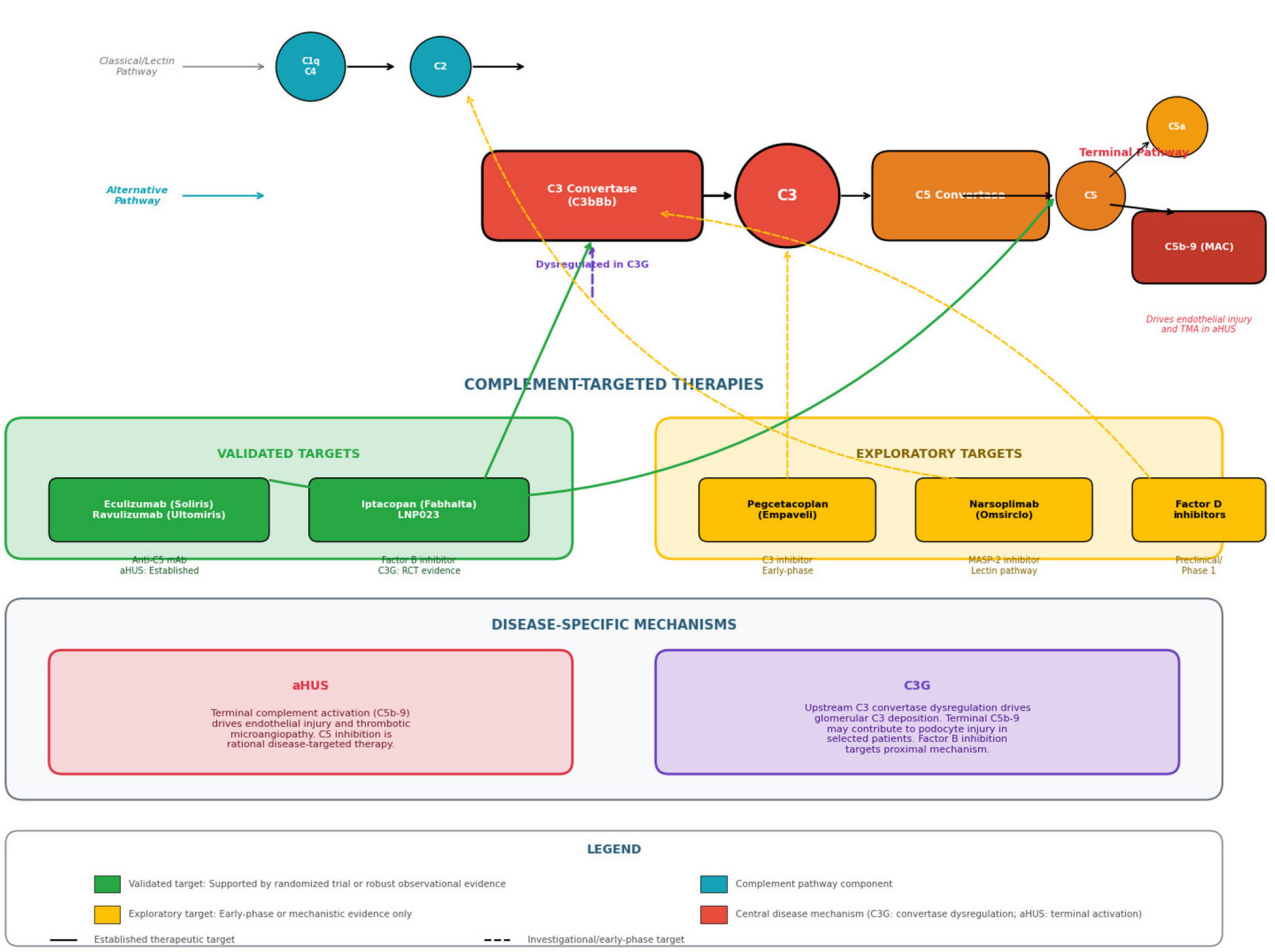
Mechanistic targets of complement-directed therapies in C3G and aHUS (Vector graphic provided as separate file.). The diagram illustrates the complement cascade with therapeutic targets: C5 inhibitors (eculizumab, ravulizumab), C3 inhibitor (pegcetacoplan), factor B inhibitor (iptacopan), factor D inhibitors, and MASP-2 inhibitor (narsoplimab). Arrow thickness and style indicate evidence strength: thick solid arrows indicate support from randomized or consistent observational evidence; thin dashed arrows indicate limited/heterogeneous evidence. For aHUS, the terminal pathway (C5b-9) arrow is thick solid (consistent observational evidence for anti-C5 therapy). For C3G, the alternative pathway C3 convertase arrow is thick solid (supported by the APPEAR-C3G RCT for factor B inhibition), whereas the terminal pathway arrow is thin dashed (heterogeneous evidence for anti-C5 therapy). Arrow style reflects the authors' qualitative synthesis of evidence volume and consistency, not a formal meta-analytic weighting. Color coding: red – terminal inhibitors; blue – proximal alternative pathway inhibitors; green – lectin pathway inhibitors.

The difference between aHUS and C3G is biologically plausible. In aHUS, terminal complement activation directly contributes to endothelial injury and TMA, making C5 inhibition a rational therapy. In C3G, the dominant abnormality is dysregulated C3 convertase activity and glomerular C3 deposition; terminal blockade may therefore be insufficient for many patients because C3 convertase overactivity can persist independently of C5 cleavage, continuing to drive C3b deposition and glomerular inflammation. This mechanistic distinction is illustrated in Figure 2: for aHUS, the terminal pathway arrow is thick and solid (consistent observational evidence for anti-C5 therapy); for C3G, the alternative pathway C3 convertase arrow is thick and solid (supported by APPEAR-C3G and VALIANT RCTs for proximal inhibition), whereas the terminal pathway arrow is thin and dashed (heterogeneous evidence for anti-C5 therapy).

### 4.3 Evidence certainty and clinical implications

GRADE assessment suggested low certainty for the effect of anti-C5 therapy on haematologic remission in aHUS because the evidence derived entirely from non-randomized studies (Table 4). For eculizumab in C3G, certainty was very low. For iptacopan in C3G, certainty was low for short-term proteinuria reduction (downgraded for short follow-up, surrogate endpoint, and indirectness regarding long-term kidney survival). For pegcetacoplan in C3G, the published VALIANT trial provides moderate-certainty evidence for short-term proteinuria reduction, though long-term kidney survival data remain pending. The cautious language (“is associated with”, “indicates”) reflects methodological limitations, not skepticism about biological plausibility.

Our findings are broadly consistent with KDIGO guidance, which recommends eculizumab or ravulizumab as first-line therapy for complement-mediated aHUS based on observational evidence (18). The KDIGO Controversies Conference emphasized comprehensive genetic work-up, complement biomarker assessment, and the distinction between proximal and terminal complement blockade in guiding patient selection. For C3G, KDIGO recommends individualized treatment with consideration of histologic activity, genetic profile, and biomarker status (18).

For aHUS, early recognition and timely complement inhibition remain central. Eculizumab and ravulizumab are both indicated for long-term treatment; discontinuation should only be considered in selected patients with resolved anti-CFH antibodies or MCP/CD46 mutations, under close monitoring. For C3G, anti-C5 therapy may be considered in selected patients with active disease and evidence of terminal pathway activation, but expectations should be cautious. Proximal inhibitors (iptacopan, pegcetacoplan) are more mechanistically aligned with C3G pathophysiology. Vaccination and infection prophylaxis are essential for all complement inhibitors.

Clinicians should recognize that the recommendation for anti-C5 therapy in aHUS rests on observational evidence and biological plausibility, not on high-certainty randomized data.

### 4.4 Limitations

This review has several limitations inherent to its rapid, time-limited design. First, the 16-day search window and rapid registration on the same day as search initiation trade comprehensiveness for timeliness. We chose this abbreviated timeframe because rapid reviews are explicitly designed to provide timely evidence for urgent clinical decisions, and the recent FDA approvals of iptacopan (March 2025) and pegcetacoplan (July 2025) created an immediate need for synthesized clinical guidance. While a sensitivity analysis identified no additional eligible studies, time-lag bias cannot be fully excluded. Second, the *post hoc* inclusion of the APPEAR-C3G and VALIANT trials, though methodologically deviant from the registered protocol, was clinically necessary because these pivotal trials were published during our review period and directly informed the regulatory landscape. Excluding them would have rendered the review obsolete. We transparently document this deviation in Supplementary Material 1. Third, the evidence base is dominated by observational studies and small case series. Fourth, outcome definitions varied substantially. Fifth, genetic panels and biomarker assays differed across studies. Sixth, Chinese databases were searched *post hoc* but no eligible studies were found; this represents a deviation from the registered protocol. Seventh, publication bias toward positive results is possible. Eighth, follow-up for emerging therapies is short. Ninth, we did not systematically evaluate post-transplant recurrence. Tenth, conference abstracts were excluded, which may have omitted negative results. Eleventh, as a rapid review, we did not perform dual independent data extraction for all studies; this may increase the risk of extraction errors. Twelfth, the GRADE certainty-of-evidence assessments were conducted on the basis of narrative synthesis rather than pooled estimates. Without meta-analytic summaries, judgements of inconsistency rely on qualitative visual inspection of study-level results rather than on statistical heterogeneity measures (e.g., I^2^, τ^2^), and judgements of imprecision rely on study-level confidence intervals and optimal information size considerations rather than on pooled confidence intervals. This introduces greater subjectivity into the certainty ratings, particularly when evaluating whether effect estimates are consistent across studies and whether confidence intervals exclude clinically meaningful benefits or harms. Readers should be aware of this limitation when interpreting the GRADE assessments. Thirteenth, while the VALIANT trial is now published in NEJM, we did not incorporate its data into primary evidence tables because the trial was identified after our search window closed; this *post hoc* inclusion is documented in Supplementary Material 1.

### 4.5 Future research

Future research should prioritize standardized outcome definitions, prospective biomarker-guided trials, and head-to-head comparisons of proximal vs. terminal inhibition. Adaptive platform trial designs and international registries with standardized data elements are needed. In aHUS, prospective discontinuation protocols incorporating genotype and biomarkers are required. In C3G, long-term kidney survival data from the APPEAR-C3G extension study and VALIANT follow-up will be critical. Patient-reported outcomes should be incorporated. The frequency and risk factors for breakthrough meningococcal infection despite vaccination warrant systematic study.

## 5 Conclusion

Based on this time-limited rapid review of available evidence, anti-C5 therapy in complement-mediated aHUS is associated with haematologic remission and renal recovery in many patients. Despite low-certainty evidence from non-randomized studies, the consistency of observed associations across multiple cohorts supports current guideline recommendations. In C3G, evidence for terminal complement blockade remains weak, whereas proximal pathway inhibition with iptacopan and pegcetacoplan has emerging randomized evidence for short-term proteinuria reduction. Biomarker-guided patient selection and standardized outcomes should be priorities. This review employed narrative synthesis without meta-analysis; readers should not interpret reported percentages as pooled estimates.

## Supplementary material

Supplementary Material 1 Full search strategies, search dates, PROSPERO registration record, PRISMA 2020 checklist, sensitivity analysis log (January-December 2025), and documentation of *post hoc* inclusion of APPEAR-C3G and VALIANT data.

Supplementary Material 2 Complete data extraction table for all 38 included studies (author, year, design, population, intervention, outcomes, risk-of-bias rating, funding).

Supplementary Material 3 Full GRADE evidence profile with domain-level reasoning, alternative grading scenarios, and comparison with guideline recommendations.

Supplementary Material 4 Vaccination and antimicrobial prophylaxis protocols for complement inhibitor therapy (MenACWY and MenB schedules, pediatric considerations).

Supplementary Material 5 List of excluded full-text studies with reasons for exclusion.

Supplementary Material 6 Chinese database (CNKI, WanFang) *post hoc* supplementary search log (search strategy, number of records, screening results, translation methods) and sensitivity analysis for studies published January-December 2025.

Supplementary Material 7 Complete 18–55 reference list in Vancouver format.
