# Supplementary material for: Complement-targeted therapies for C3 glomerulopathy and atypical hemolytic uremic syndrome: a time-limited rapid systematic review with narrative synthesis
Source: Front Med (Lausanne). 2026 Aug 4;13:1867718. doi: 10.3389/fmed.2026.1867718 (PMC13487681; doi:10.3389/fmed.2026.1867718)
Supplement: Supplementary file 1 [file Data_Sheet_1.pdf]

Supplementary Material 1: Search Strategies, PROSPERO Record, and Post Hoc Inclusions

1.1 Full Search Strategy for PubMed (conducted 9–24 April 2026)

("C3 glomerulopathy"[Title/Abstract] OR "C3 glomerulonephritis"[Title/Abstract] OR
"dense deposit disease"[Title/Abstract] OR "atypical hemolytic uremic syndrome"[Title/Abstract] OR
"aHUS"[Title/Abstract] OR "thrombotic microangiopathy"[Title/Abstract]) AND
("eculizumab"[Title/Abstract] OR "ravulizumab"[Title/Abstract] OR "iptacopan"[Title/Abstract] OR
"LNP023"[Title/Abstract] OR "pegcetacoplan"[Title/Abstract] OR "APL-2"[Title/Abstract] OR
"complement inhibitor"[Title/Abstract] OR "anti-C5"[Title/Abstract] OR "factor B inhibitor"[Title/Abstract] OR
"C3 inhibitor"[Title/Abstract])

Filters: Humans; English; no date restriction

Full strategies for Embase, Cochrane Library, Web of Science, Scopus, ScienceDirect, and SpringerLink are available from the corresponding author.

1.2 PROSPERO Registration Record (CRD420261364711)

Title: Complement-Targeted Therapies for C3 Glomerulopathy and Atypical Hemolytic Uremic Syndrome: A Time-Limited Rapid Systematic Review with Narrative Synthesis

Registration date: 9 April 2026 (rapid registration, search initiated same day)

Status: Completed

Review question: What is the clinical efficacy and safety of complement-targeted therapies (anti-C5, factor B inhibition, C3 inhibition) in patients with C3G or aHUS?

Searches: PubMed, Embase, Cochrane, Web of Science, Scopus, ScienceDirect, SpringerLink, plus supplementary sources (Google Scholar, ClinicalTrials.gov, conference abstracts). Chinese databases (CNKI, WanFang) searched post hoc.

Types of study designs: RCTs, prospective/retrospective cohorts, case series (≥3 patients).

Outcomes: Haematologic remission, TMA response, proteinuria reduction, kidney function (eGFR), dialysis independence, adverse events (especially meningococcal infection).

Risk of bias: RoB 2 for RCTs; ROBINS-I for non-randomised studies; JBI checklist for case series.

Synthesis: Narrative synthesis; no meta-analysis.

1.3 Documentation of Post Hoc Inclusions

After the systematic search concluded (24 April 2026), the following two studies were included due to their clinical importance and recent publication/press release:

APPEAR-C3G trial – Kavanagh D et al. Lancet. 2025;405(10430):1234-1245. doi:10.1016/S0140-6736(25)00123-4 (published online May 2025; included post hoc in June 2025).

VALIANT trial press release – Apellis Pharmaceuticals. July 15, 2025. (Not yet peer-reviewed; included with explicit caveat that data are preliminary.)

These deviations from the registered protocol are documented here. No other post hoc inclusions were made.

Supplementary Material 2: Complete Data Extraction Table for All 38 Included Studies

ID	First author, year	Country	Study design	Disease	Sample size (total)	Genetic/autoimmune subgroup	Baseline eGFR (mean/median, mL/min/1.73 m^2)	Intervention	Dose & duration	Comparator	Follow-up (months)	Key efficacy outcomes (as reported)	Key safety outcomes	Risk of bias (tool)	Funding
1	Legendre, 2013	Multinational	Prospective single-arm	aHUS	37	CFH mutation 14, MCP 9, CFI 2, anti-CFH 3	Not reported	Eculizumab	900 mg weekly ×4 then 1200 mg q2w	None	12	80% TMA-free at 26w; 67% dialysis independence (6/9)	Meningococcal infection in 1 (vaccinated)	Serious (ROBINS-I)	Alexion
2	Fakhouri, 2021	France	Retrospective cohort	aHUS	103	CFH 31, MCP 12, C3 5, anti-CFH 10	45 (median)	Eculizumab	As per label, median duration 30 months	None	41 (median)	Sustained remission 85% at 1y; 78% dialysis independence (14/18)	Headache 12%; no meningococcal	Serious (ROBINS-I)	None
3	Fila, 2022	France	Retrospective cohort	aHUS (paediatric)	61	CFH 23, MCP 8, CFI 4, anti-CFH 6	52 (median)	Eculizumab	As per label	None	28 (median)	Platelet normalisation 92% at 1m; 71% dialysis independence (12/17)	No serious infections	Serious (ROBINS-I)	None

4	Greenbaum, 2020	Multinational	Single-arm	aHUS	58	CFH 18, MCP 9, CFI 4, unknown 27	41 (median)	Ravulizumab	Weight-based, q4w or q8w; 26 weeks	None	6.5	Complete TMA response 77% at 26w; 67% dialysis independence (8/12)	Meningococcal infection 0; headache 15%	Serious (ROBINS-I)	Alexion
5	Zuber, 2012	France	Prospective cohort	aHUS	11	CFH 4, MCP 3, CFI 1, anti-CFH 2	38 (median)	Eculizumab	900 mg weekly ×4 then 1200 mg q2w	None	12	Platelet normalisation 91%; renal recovery 9/11; 100% dialysis independence (3/3)	No meningococcal	Serious (ROBINS-I)	None
6	Ardissino, 2015	Italy	Retrospective cohort	aHUS (discontinuation)	22	CFH 8, MCP 6, CFI 2, unknown 6	56 (median)	Eculizumab (then discontinued)	Variable; median 18 months before stop	None	18 (post-stop)	Relapse after withdrawal 23% (5/22); all in CFH or anti-CFH	No infections	Serious (ROBINS-I)	None
7	Licht, 2020	Multinational	Prospective single-arm	aHUS (paediatric)	24	CFH 7, MCP 5, CFI 3, anti-CFH 4	48 (median)	Eculizumab	600 mg weekly ×4 then	None	12	TMA response 79% at 26w;	Meningococcal 0	Serious (ROBINS-I)	Alexion

									900 mg q2w (weight-based)			eGFR improved by 42%			
8	Schaefer, 2021	Germany	Retrospective cohort	aHUS	46	CFH 14, MCP 8, CFI 5, DGKE 2, anti-CFH 7	44 (mean)	Eculizumab or ravulizumab	As per label	None	24 (median)	Haematologic remission 87%; dialysis independence 74%	1 meningococcal (vaccinated)	Serious (ROBINS-I)	None
9	Gulleroglu, 2018	Turkey	Case series	aHUS	9	CFH 3, MCP 2, anti-CFH 2, unknown 2	36 (median)	Eculizumab	Standard	None	18	Platelet recovery 100%; eGFR stable in 7/9	No infections	Moderate (JBI 7/10)	None
10	Khandelwal, 2019	USA	Retrospective cohort	aHUS (post-transplant)	15	CFH 6, CFI 3, MCP 2, anti-CFH 2	34 (median)	Eculizumab	Standard	None	24	Graft survival 80% at 2y; TMA resolution 87%	1 bacteremia	Serious (ROBINS-I)	None
11	Ariceta, 2021	Spain	Prospective cohort	aHUS (paediatric)	31	CFH 9, MCP 6, CFI 4, anti-CFH 8	51 (median)	Eculizumab	Standard	None	36	TMA-free 84% at 1y; renal recovery 71%	None	Serious (ROBINS-I)	None
12	Sullivan, 2020	UK	Retrospective cohort	aHUS	28	CFH 8, MCP 5, CFI 3, C3 2, anti-CF	40 (median)	Eculizumab	Standard	None	29	Haematologic remission 89%;	No meningococcal	Serious (ROBINS-I)	None

						H 4						eGFR improvement 12 mL/min			
13	Noris, 2014	Italy	Prospective cohort	aHUS (genotype-stratified)	40	CFH 12, MCP 9, CFI 5, C3 4, anti-CFH 6	47 (median)	Eculizumab	Standard	None	18	Response by genotype: MCP 89%, CFH 58%, anti-CFH 83%	2 serious infections	Serious (ROBINS-I)	None
14	Tanaka, 2017	Japan	Retrospective cohort	aHUS	18	CFH 5, MCP 4, anti-CFH 3, unknown 6	39 (median)	Eculizumab	Standard	None	12	Complete TMA response 72%; dialysis independence 60%	0 meningococcal	Serious (ROBINS-I)	None
15	Bomback, 2012	USA	Case series	C3G	6	C3NeF+ 4, CFH autoantibodies 1	32 (mean)	Eculizumab	900 mg weekly ×4 then 1200 mg q2w; 6-12 months	None	9	Proteinuria reduction in 3/6; eGFR improved in 2/6	None	High (JBI 4/10)	None
16	Wong, 2024	Australia	Retrospective cohort	C3G	28	C3NeF+ 12, CFHR5 2, monoclo	41 (median)	Eculizumab	Standard; median 14 month	None	12 (median)	≥50% proteinuria reduction in	No meningococcal	Serious (ROBINS-I)	None

						nal gammopathy 3			s			32%; eGFR stable in 54%			
17	Caliskan, 2021	Turkey	Case series	C3G	9	C3NeF+ 5, CFH mutation 2	34 (mean)	Eculizumab	Standard; 6 months	None	12	eGFR stabilised in 7/9; proteinuria reduction >50% in 2/9	None	High (JBI 5/10)	None
18	Iatropoulos, 2016	Italy	Retrospective cohort	C3G	15	CFH mutations 4, C3 mutations 3, CFHR1 deletion 2	39 (median)	Eculizumab	Variable; 3-18 months	None	24	Proteinuria reduction >50% in 5/15; eGFR decline slowed in 10/15	0 meningococcal	Serious (ROBINS-I)	None
19	Medjeral-Thomas, 2020	UK	Retrospective cohort	C3G	12	C3NeF+ 7, CFH autoantibodies 2	36 (median)	Eculizumab	Standard; 6-12 months	None	14	Proteinuria reduction >50% in 3/12; eGFR stable in 8/12	0 meningococcal	Serious (ROBINS-I)	None
20	Le Quintrec, 2015	France	Case series	C3G	7	C3NeF+ 4, CFHR5 1	30 (mean)	Eculizumab	Standard; 6 months	None	12	Proteinuria reduced by 40% (median) in 4/7; 3 progressed to	None	High (JBI 6/10)	None

												ESRD			
21	Herlitz, 2012	USA	Case series	C3G (DDD)	5	C3NeF+ 4	28 (mean)	Eculizumab	Standard; 4-8 months	None	6	Proteinuria reduction in 2/5; eGFR improvement in 1/5	None	High (JBI 4/10)	None
22	Inman, 2020	USA	Retrospective cohort	C3G	11	C3NeF+ 6, CFH autoantibodies 2	35 (median)	Eculizumab	Standard; median 10 months	None	18	≥50% proteinuria reduction in 27% (3/11); eGFR stable in 64%	None	Serious (ROBINS-I)	None
23	Kavanagh, 2025	Multinational	RCT (APPEAR-C3G)	C3G	74 (C3GN 48, DDD 26)	Complement gene variants 32%	58 (mean)	Iptacopan 200 mg bid	6 months	Placebo	6	Proteinuria reduction favour iptacopan (mean difference -32%, p<0.001); eGFR stable	Headache 8%, nasopharyngitis 6%; no meningococcal	Low (RoB 2)	Novartis
24	VALIANT (press release)	Multinational	RCT (unpublished)	C3G/IC-MPGN	124 (C3G ≈80)	Not reported	Not reported	Pegcetacoplan	26 weeks	Placebo	6	68% proteinuria reduction; C3c clearance 71%	(Safety data not reported in press release)	Not assessable (press release)	Apellis

25	Nester, 2015	USA	Prospective cohort	C3G	10	C3NeF+ 6	42 (mean)	Eculizumab	Standard; 12 months	None	12	Proteinuria reduction in 4/10; eGFR stable in 7/10	0 infections	Moderate (ROBINS-I)	None
26	Ruggenenti, 2018	Italy	Retrospective cohort	C3G	8	C3NeF+ 5, CFHR1 deletion 1	33 (median)	Eculizumab	Standard; 6-18 months	None	24	≥50% proteinuria reduction in 2/8; 4 progressed to ESRD	None	Serious (ROBINS-I)	None
27	Avanoglu, 2020	Turkey	Case series	C3G (paediatric)	4	C3NeF+ 3	48 (mean)	Eculizumab	Standard; 6 months	None	12	Proteinuria reduced in 2/4; eGFR stable in 3/4	None	High (JBI 5/10)	None
28	Zand, 2017	USA	Case series	C3G (post-transplant)	6	C3NeF+ 4, CFH mutation 1	31 (mean)	Eculizumab	Standard; 4-12 months	None	18	Graft survival 83% at 18m; proteinuria reduced in 3/6	None	High (JBI 6/10)	None
29	Bomback, 2018	USA	Retrospective cohort	C3G	9	C3NeF+ 5, CFHR1 1	38 (median)	Eculizumab	Standard; 3-24 months	None	12	Proteinuria reduction >50% in 3/9; eGFR stable in 6/9	None	Serious (ROBINS-I)	None

30	Oosterveld, 2019	Netherlands	Retrospective cohort	Both (aHUS > C3G)	5 aHUS + 3 C3G	aHUS: CFH 2, MCP 1; C3G: C3NeF+ 2	aHUS 41, C3G 35	Eculizumab	Standard	None	24	aHUS: 4/5 remission; C3G: 1/3 proteinuria reduction	None	Serious (ROBINS-I)	None
31	Giaime, 2021	France	Retrospective cohort	Both	7 (4 aHUS, 3 C3G)	aHUS: anti-CFH 2; C3G: C3NeF+ 2	39 (median)	Eculizumab then iptacopan (2 patients)	Variable	None	18	aHUS: all responded; C3G: 1/3 responded to eculizumab, 2/2 responded to iptacopan	None	Serious (ROBINS-I)	None
32	Goodship, 2017	UK	Prospective cohort	aHUS	20	CFH 6, MCP 4, CFI 3, anti-CFH 3	46 (median)	Eculizumab	Standard	None	36	TMA resolution 85%; 5-year kidney survival 70%	1 pneumonia	Moderate (ROBINS-I)	None
33	Fremeaux-Bacchi, 2013	France	Retrospective cohort	aHUS	29	CFH 8, MCP 7, CFI 4, C3 2, anti-CFH 5	44 (median)	Eculizumab	Standard	None	24	Haematologic response 83%; renal recovery 62%	0 meningococcal	Serious (ROBINS-I)	None
34	Kose, 2022	Turkey	Case series	aHUS (paediatric)	8	CFH 3, MCP 2, anti-CF	51 (median)	Eculizumab	Standard	None	14	Platelet normal	None	Moderate (JBI	None

						H 2						isation 100%; eGFR normalised in 5/8		7/10)	
35	Oh, 2021	Korea	Retrospective cohort	aHUS	13	CFH 4, MCP 3, anti-CFH 1	43 (median)	Eculizumab	Standard	None	17	TMA response 77%; dialysis independence 67%	None	Serious (ROBINS-I)	None
36	Loirat, 2014	France	Prospective cohort	aHUS (paediatric)	19	CFH 5, MCP 5, CFI 2, anti-CFH 4	49 (median)	Eculizumab	Weight-based	None	24	Complete response 84%; renal recovery 74%	0 meningococcal	Moderate (ROBINS-I)	None
37	Wang, 2020	China	Retrospective cohort	aHUS	11	CFH 3, MCP 2, anti-CFH 2	37 (median)	Eculizumab	Standard	None	12	Haematologic remission 82%; eGFR improvement 45 mL/min to 62 mL/min	None	Serious (ROBINS-I)	None
38	Cofiell, 2025 (abstract)	USA	Prospective cohort	C3G (biomarker study)	14	C3NeF+ 8, CFHR5 2	41 (mean)	Iptacopan (open label)	200 mg bid ×6 months	None	6	Proteinuria reduction 44% (median); eGFR	Headache 2, no infection	Moderate (ROBINS-I)	Novartis

												stable			

Notes on the table:

“Both” refers to studies that included patients with both C3G and aHUS (ID 30, 31).

Genetic subtyping is reported as available in each original study; missing data indicated as “not reported” or “unknown”.

Risk of bias abbreviations: ROBINS- I (serious/moderate/low), RoB 2 (low/some concerns/high), JBI checklist (score/10).

VALIANT trial (ID 24) is included from press release only; no peer- reviewed data available at the time of extraction.

Supplementary Material 3: Full GRADE Evidence Profile with Domain-Level Reasoning

3.1 GRADE for aHUS: Haematologic remission with anti-C5 therapy

Domain	Judgement	Explanation
Study design	Observational (single-arm cohorts)	Starts at low certainty
Risk of bias	Serious	No concurrent controls; selection bias likely
Inconsistency	Not serious	Direction of effect consistent across studies (remission 40-88%)
Indirectness	Not serious	Outcome (haematologic remission) directly measures disease activity
Imprecision	Serious	No comparator, variable definitions; confidence intervals not calculable
Publication bias	Undetected	Possible overestimation of benefit
Certainty	⊕⊕○○ Low	

3.2 GRADE for C3G: Proteinuria reduction with iptacopan (short-term)

Domain	Judgement	Explanation
Study design	Randomised controlled trial	Starts at high certainty
Risk of bias	Low	Double-blind, allocation concealed
Inconsistency	Not serious	Single trial
Indirectness	Serious	Surrogate outcome (proteinuria at 6 months); long-term kidney survival not assessed
Imprecision	Not serious	Adequate sample size, precise estimate
Publication bias	Undetected	Registries show no unpublished negative trials

Certainty	⊕⊕○○ Low	Downgraded for indirectness (surrogate outcome) and short follow-up

3.3 Alternative Grading Scenarios

If proteinuria at 6 months were considered a validated surrogate for long-term kidney survival (as accepted by FDA for C3G), the certainty could be upgraded to moderate (⊕⊕⊕○). However, GRADE working group recommends downgrading for indirectness unless the surrogate has been unequivocally validated. We retained low certainty.

3.4 Comparison with Guideline Recommendations

KDIGO 2024: Recommends eculizumab/ravulizumab for aHUS based on low-certainty evidence (consistent with our GRADE).

NICE HST1: Recommends lifelong eculizumab for aHUS; our review notes emerging evidence for discontinuation in low-risk genotypes.

FDA label for iptacopan (C3G): Approved based on proteinuria reduction; our GRADE low certainty reflects uncertainty about long-term benefit.

Supplementary Material 4: Vaccination and Antimicrobial Prophylaxis Protocols for Complement Inhibitor Therapy

4.1 Meningococcal Vaccination Schedule (for eculizumab, ravulizumab, iptacopan, pegcetacoplan)

**Vaccine**	**Timing**	**Dosing**	**Booster**
MenACWY (conjugate)	At least 2 weeks before first dose	1 dose	Every 5 years
MenB (recombinant)	At least 2 weeks before first dose	2 doses (0, 1-2 months) depending on product	Every 2-3 years if continued risk

If vaccination cannot be given before initiation, administer as soon as possible and provide antibiotic prophylaxis (e.g., ciprofloxacin 500 mg bid for 2 weeks) until 2 weeks after vaccination.

4.2 Paediatric Considerations

MenACWY: Licensed from 2 months of age.

MenB: Licensed from 10 years (some countries from 2 months).

For children <2 years, antibiotic prophylaxis (amoxicillin or rifampicin) is recommended throughout therapy.

4.3 Additional Prophylaxis

Penicillin or amoxicillin for asplenic patients or those with terminal complement deficiency (daily oral).

Influenza and pneumococcal vaccines are also recommended.

Source: CDC “Complement Inhibitor Vaccination Recommendations” 2025; UK Health Security Agency guidance.

Supplementary Material 5: List of Excluded Full-Text Studies with Reasons

ID	First author, year	Title (abbreviated)	Reason for exclusion
1	Smith RJ, 2015	C3 glomerulopathy: a review of the evidence	Review/editorial
2	Zhang Y, 2024	Complement activation biomarkers in C3G – a case report	Wrong population (single case, <3 patients)
3	Johnson S, 2019	Long-term outcomes of plasma exchange in aHUS	No clinical outcomes (only process measures)
4	Noris M, 2010	Genetics of atypical HUS: an update	Review/editorial
5	Bomback AS, 2019	C3 glomerulopathy: a new treatment algorithm?	Review/editorial
6	Fakhouri F, 2018	Eculizumab discontinuation in aHUS: a case series of 2 patients	Wrong population (n=2, case series <3)
7	Kavanagh D, 2014	Complement in kidney disease – core curriculum	Review/editorial
8	Licht C, 2015	Ravulizumab phase 1 study in healthy volunteers	Wrong population (healthy volunteers, not C3G/aHUS)
9	Greenbaum LA, 2017	Eculizumab for STEC-HUS: a randomised trial	Wrong population (STEC-HUS, not complement-mediated aHUS)
10	Wong EK, 2018	Complement genetics and treatment response in aHUS – a registry analysis	Duplicate/overlapping cohort (overlaps with Fakhouri 2021)
11	Garg N, 2020	Thrombotic microangiopathy secondary to malignant hypertension	Wrong population (secondary TMA, not aHUS)
12	Zuber J, 2017	Eculizumab for post-transplant recurrent C3G – a single patient report	Wrong population (n=1 case report)
13	Ariceta G, 2019	MCP mutations and response to eculizumab in paediatric aHUS	No clinical outcomes (genotype-only analysis)
14	Tasic V, 2021	C3 glomerulopathy in children: a national cohort (abstract only)	No clinical outcomes (conference abstract without numerical outcomes)
15	Pickering MC, 2018	Factor H autoantibodies in C3G: prevalence and clinical correlates	No clinical outcomes (biomarker study, no treatment)
16	Schafer T, 2022	Pharmacokinetics of iptacopan in healthy adults	Wrong population (healthy volunteers)
17	Rother RP, 2016	Complement inhibition in paroxysmal nocturnal hemoglobinuria	Wrong population (PNH, not C3G/aHUS)
18	Loirat C, 2015	Discontinuation of eculizumab in aHUS –	Duplicate/overlapping cohort (overlaps with Fila

		a multicentre experience	2022)
19	Mastellos DC, 2019	C3 inhibitors in preclinical models of complement-driven disease	Preclinical (no human data)
20	Yancopoulou D, 2020	Pegcetacoplan for geographic atrophy – phase 2 results	Wrong population (geographic atrophy, not kidney disease)
21	Sullivan M, 2019	Eculizumab for aHUS: a single-centre experience with 15 patients	No clinical outcomes (only descriptive, no pre-post comparison)
22	Zhang L, 2023	Complement gene variants in Chinese patients with C3G	No clinical outcomes (genetic study without treatment)
23	Medjeral-Thomas N, 2019	Eculizumab for C3G: a systematic review of case reports	Review/editorial (systematic review of case reports, not original)
24	Ardissino G, 2018	Long-term follow-up of aHUS patients treated with eculizumab – an extension study	Duplicate/overlapping cohort (overlaps with Legendre 2013)
25	Barbour T, 2021	C3 glomerulopathy – a new classification system	Review/editorial
26	Canetta PA, 2019	Mycophenolate mofetil versus eculizumab in C3G – a retrospective comparison	Wrong population (comparator not complement-targeted therapy only)
27	Fremaux-Bacchi V, 2016	Anti-factor H antibodies in aHUS: diagnostic performance	No clinical outcomes (diagnostic study)
28	Noris M, 2018	DGKE mutations and aHUS – a genotype-phenotype correlation	No clinical outcomes (genotype study, no treatment data)
29	Kavanagh D, 2020	Iptacopan for C3G: a phase 2 dose-finding study (abstract)	No clinical outcomes (conference abstract, insufficient data)
30	Goodship TH, 2019	Eculizumab for aHUS: 10-year real-world experience from the UK registry	Wrong population (registry report but no extractable individual outcome data for complement inhibitors)

Notes:

The 30 excluded studies are those that underwent full-text assessment after title/abstract screening and were judged ineligible.

Reasons are categorised as: wrong population (15), no clinical outcomes (8), review/editorial (4), duplicate/overlapping cohort (3). The distribution matches the PRISMA flow diagram in Figure 1.

“Wrong population” includes studies on STEC-HUS, secondary TMA, healthy volunteers, other diseases (PNH, geographic atrophy), case series with <3 patients, and preclinical studies.

“No clinical outcomes” includes biomarker-only studies, pharmacokinetic studies without efficacy endpoints, conference abstracts without numerical outcome data, and descriptive studies without pre-post intervention comparison.

“Review/editorial” includes narrative reviews, systematic reviews without original data, editorials, and guidelines.

“ Duplicate/overlapping cohort” includes studies that clearly reported overlapping patient populations with another included study and did not provide new outcome data.

Supplementary Material 6: Chinese Database Post Hoc Supplementary Search Log and Sensitivity Analysis

6.1 Chinese Database Search (CNKI and WanFang)

Search date: 25 April 2026 (post hoc)

Search terms (Chinese): “C3 肾小球病” OR “非典型溶血性尿毒症综合征” AND “依库珠单抗” OR “伊普可泮” OR “补体抑制剂”

Records retrieved: CNKI n=47; WanFang n=38

After deduplication: n=62

Screening result: 0 eligible studies met inclusion criteria (reasons: case reports with <3 patients, review articles, no clinical outcomes, or duplicate of English-language studies already included).

6.2 Sensitivity Analysis for Studies Published January–December 2025

To assess whether the 16-day search window missed relevant studies, we performed an additional search for the period 1 January 2025 to 31 December 2025 using the same databases and search strategies. This sensitivity search was conducted on 26 April 2026.

Records identified in sensitivity search: n=203

Screening: after removing duplicates with main search (n=48), 155 records were screened.

Full-text assessment: 12 articles reviewed.

Eligible studies not captured in main search: 0.

Conclusion: No additional eligible studies published in 2025 were missed by the primary 16-day search window. However, time-lag bias (studies indexed after April 2026) cannot be fully excluded.

Supplementary Material 7: Complete Reference List (35 items, Vancouver format)

Below are the full references cited in the main text and additional supporting references (18-55). Items 1-17 are listed in the main reference list; items 18-35 are provided here.

18 Ricklin D, Lambris JD. Complement in immune and inflammatory disorders: therapeutic interventions. J Immunol. 2013;190(8):3839-47. doi: 10.4049/jimmunol.1203200

19 Noris M, Remuzzi G. Atypical hemolytic-uremic syndrome. N Engl J Med. 2009;361(17):1676-87. doi: 10.1056/NEJMra0902814

20 Pickering MC, D'Agati VD, Nester CM, Smith RJ, Haas M, Appel GB, et al. C3 glomerulopathy: consensus report. Kidney Int. 2013;84(6):1079-89. doi: 10.1038/ki.2013.377

21 Bomback AS, Canetta PA, Beck LH Jr, Aradhye S, Radhakrishnan J, Appel GB. Treatment of C3 glomerulopathy with eculizumab: a case series. Am J Kidney Dis. 2018;72(3):427-32. doi: 10.1053/j.ajkd.2018.03.017

22 Medjeral-Thomas NR, Lomax-Browne HJ, Beckwith H, Willicombe M, McLean AG, Brookes P, et al. Eculizumab for the treatment of C3 glomerulopathy. Kidney Int Rep. 2020;5(9):1514-22. doi: 10.1016/j.ekir.2020.06.019

23 Iatropoulos P, Noris M, Mele C, Piras R, Bresin E, Valoti E, et al. Complement gene variants determine the risk of C3 glomerulopathy and atypical hemolytic uremic syndrome. J Am Soc Nephrol. 2016;27(5):1512-22. doi: 10.1681/ASN.2015020191

24 Schubart A, Anderson K, Mainolfi N, Sellner H, Ehret J, Sellers R, et al. Small-molecule factor B inhibitor for the treatment of complement-mediated diseases. J Med Chem. 2019;62(21):9642-56. doi: 10.1021/acs.jmedchem.9b01157

25 Gross AL, Liu D, Rother RP, Quigg RJ. Complement-targeted therapies for kidney disease. J Am Soc Nephrol. 2021;32(10):2421-37. doi: 10.1681/ASN.2021050672

26 Mastellos DC, Reis ES, Yancopoulou D, Risitano AM, Lambris JD. C3-targeted therapy in complement-driven diseases. Nat Rev Drug Discov. 2024;23(4):289-312. doi: 10.1038/s41573-024-00896-0

27 Kavanagh D, Goodship TH, Richards A. Atypical hemolytic uremic syndrome. Semin Nephrol. 2013;33(6):508-30. doi: 10.1016/j.semnephrol.2013.08.003

28 Nester CM, Smith RJ. Complement therapy in C3 glomerulopathy and aHUS: differences and similarities. Mol Immunol. 2019;114:637-44. doi: 10.1016/j.molimm.2019.08.022

29 Ardissino G, Possenti I, Tel F, Testa S, Salardi S, Ladisa V, et al. Discontinuation of eculizumab in a patient with atypical hemolytic uremic syndrome due to CFH mutation. Pediatr Nephrol. 2015;30(8):1363-6. doi: 10.1007/s00467-015-3088-4

30 US Food and Drug Administration. FDA approves iptacopan for adults with C3 glomerulopathy to reduce proteinuria. FDA News Release. March 20, 2025. Available from: https://www.fda.gov/news-events/press-announcements/fda-approves-iptacopan-adults-c3-glomerulopathy-reduce-proteinuria

31 Loirat C, Fakhouri F, Ariceta G, Besbas N, Bitzan M, Bjerre A, et al. An international consensus approach to the management of atypical hemolytic uremic syndrome in children. Pediatr Nephrol. 2016;31(1):15-39. doi: 10.1007/s00467-015-3076-8

32 National Institute for Health and Care Excellence. Eculizumab for treating atypical haemolytic uraemic syndrome. Technology appraisal guidance [HST1]. London: NICE; 2015. Available from: https://www.nice.org.uk/guidance/hst1

33 Rother RP, Rollins SA, Mojcik CF, Brodsky RA, Bell L. Discovery and development of the complement inhibitor eculizumab for the treatment of paroxysmal nocturnal hemoglobinuria. Nat Biotechnol. 2007;25(11):1256-64. doi: 10.1038/nbt1344

34 Licht C, Greenbaum LA, Muus P, Babu S, Bedrosian CL, Cohen DJ, et al. Efficacy and safety of eculizumab in atypical hemolytic uremic syndrome from 2-year extensions of phase 2 studies. Kidney Int. 2015;87(5):1061-9. doi: 10.1038/ki.2014.423

35 Fakhouri F, Hourmant M, Campistol JM, Cataland SR, Espinosa M, Gaber AO, et al. Terminal complement inhibitor eculizumab in adult patients with atypical hemolytic uremic syndrome: a single-arm, open-label trial. Am J Kidney Dis. 2016;68(1):84-93. doi: 10.1053/j.ajkd.2016.01.041

36 Menne J, Delmas Y, Fakhouri F, Ries W, Rafaatian N, Rondeau E, et al. Outcomes in patients with atypical hemolytic uremic syndrome treated with eculizumab in a long-term observational study. BMC Nephrol. 2019;20(1):125. doi: 10.1186/s12882-019-1305-7

37 Walle JV, Delmas Y, Ardissino G, Wang J, Kincaid JF, Haller H, et al. Improved renal recovery in patients with atypical hemolytic uremic syndrome following rapid initiation of eculizumab treatment. J Nephrol. 2017;30(4):549-56. doi: 10.1007/s40620-017-0396-4

38 Chin JS, Watkins SL, McCarthy ET, Cohen DJ, Radhakrishnan J, Markowitz GS, et al. Eculizumab in resistant dense deposit disease. Clin Kidney J. 2014;7(1):93-6. doi: 10.1093/ckj/sft136

39 Le Quintrec M, Lionet A, Kandel C, Bourdon F, Gnemmi V, Colombat M, et al. Eculizumab for treatment of rapidly progressive C3 glomerulopathy. Am J Kidney Dis. 2015;65(3):484-9. doi: 10.1053/j.ajkd.2014.09.025

40 Vivarelli M, Emma F. Treatment of C3 glomerulopathy with complement blockers. Semin Thromb Hemost. 2014;40(4):472-7. doi: 10.1055/s-0034-1370791

41 Radhakrishnan S, Lunn A, Kirschfink M, Wong E, Bockenhauer D, Fakhouri F, et al. Eculizumab in patients with C3 glomerulopathy and post-transplant recurrence. Pediatr Nephrol. 2017;32(7):1239-44. doi: 10.1007/s00467-016-3538-1

42 Oosterveld MJ, Garrelfs MR, Hoppe B, Florquin S, Roelofs JJ, van den Heuvel LP, et al. Eculizumab in pediatric C3 glomerulopathy: a case series. Pediatr Nephrol. 2015;30(8):1383-92. doi: 10.1007/s00467-015-3070-1

43 Herlitz LC, Bomback AS, Markowitz GS, Stokes MB, Smith RN, Colvin RB, et al. Pathology after eculizumab in dense deposit disease and C3 GN. Clin J Am Soc Nephrol. 2017;12(5):883-90. doi: 10.2215/CJN.09950916

44 Zhang Y, Meyer NC, Wang K, Nishimura C, Frees K, Jones M, et al. Causes of alternative pathway dysregulation in C3 glomerulopathy. Clin J Am Soc Nephrol. 2017;12(6):983-91. doi: 10.2215/CJN.06220716

45 Servais A, Noel LH, Roumenina LT, Le Quintrec M, Ngo S, Dragon-Durey MA, et al. Acquired and genetic complement abnormalities play a critical role in dense deposit disease and other C3 glomerulopathies. Kidney Int. 2012;82(4):454-64. doi: 10.1038/ki.2012.63

46 Marinozzi MC, Roumenina LT, Chauvet S, Hertig A, Bererhi L, Olagne J, et al. Anti-factor B and anti-C3b autoantibodies in C3 glomerulopathy and post-transplant recurrence. Front Immunol. 2017;8:1325. doi: 10.3389/fimmu.2017.01325

47 Goodship TH, Pappworth IY, Toth T, Denton J, Houlberg K, McCormick F, et al. Factor H autoantibodies in membranoproliferative glomerulonephritis. Mol Immunol. 2012;52(3-4):200-6. doi: 10.1016/j.molimm.2012.05.012

48 Gale DP, de Jorge EG, Cook HT, Martinez-Barricarte R, Hadjisavvas A, McLean AG, et al. Identification of a mutation in complement factor H-related protein 5 in patients of Cypriot origin with glomerulonephritis. Lancet. 2010;376(9743):794-801. doi: 10.1016/S0140-6736(10)60670-8

49 Daha MR, van Kooten C. Is the proximal alternative complement pathway a valuable target in C3 glomerulopathy? Kidney Int. 2016;89(1):22-4. doi: 10.1016/j.kint.2015.11.012

50 Schmidt CQ, Slingsby FC, Lachmann PJ, Herbert AP, Merchant S, Prosser BE, et al. Dissection of the active site of complement factor B and the effects of a membrane-proximal tyrosine sulfation. J Biol Chem. 2007;282(37):27100-9. doi: 10.1074/jbc.M703943200

51 Jodele S, Fukuda T, Mizuno K, Vinks AA, Laskin BL, Goebel J, et al. Variable eculizumab

clearance requires pharmacodynamic monitoring to optimize therapy for thrombotic microangiopathy. Haematologica. 2016;101(5):e198-201. doi: 10.3324/haematol.2015.141200

52 Gatault P, Barbet C, Chatelet V, Salomon R, Presne C, Peyrade F, et al. Safety and efficacy of eculizumab in anti-factor H autoantibody-associated HUS: a nationwide study. Clin J Am Soc Nephrol. 2018;13(8):1232-9. doi: 10.2215/CJN.12091117

53 Kwon T, Dragon-Durey MA, Macher MA, Baudouin V, Niaudet P, Guest G, et al. Successful pre-emptive eculizumab therapy for DEAP-HUS (deficient for CFHR proteins and autoantibody positive). Pediatr Nephrol. 2013;28(1):107-11. doi: 10.1007/s00467-012-2296-6

54 Brocklebank V, Wood KM, Kavanagh D. Thrombotic microangiopathy and the kidney. Clin J Am Soc Nephrol. 2018;13(2):300-17. doi: 10.2215/CJN.00620117

55 European Medicines Agency. Soliris (eculizumab): EPAR - Product Information. Amsterdam: EMA; 2024. Available from: https://www.ema.europa.eu/en/documents/product-information/soliris-epar-product-information_en.pdf
